# Introduction of the Carotenoid Biosynthesis α-Branch Into *Synechocystis* sp. PCC 6803 for Lutein Production

**DOI:** 10.3389/fpls.2021.699424

**Published:** 2021-07-06

**Authors:** Martin Lehmann, Evgenia Vamvaka, Alejandro Torrado, Peter Jahns, Marcel Dann, Lea Rosenhammer, Amel Aziba, Dario Leister, Thilo Rühle

**Affiliations:** ^1^Plant Molecular Biology, Faculty of Biology, Ludwig-Maximilians-University Munich, Munich, Germany; ^2^Plant Biochemistry, Heinrich-Heine-University Düsseldorf, Düsseldorf, Germany

**Keywords:** lutein, carotenoids, cyanobacteria, *Synechocystis*, cyclase, genetic engineering, *Arabidopsis thaliana*

## Abstract

Lutein, made by the α-branch of the methyl-erythritol phosphate (MEP) pathway, is one of the most abundant xanthophylls in plants. It is involved in the structural stabilization of light-harvesting complexes, transfer of excitation energy to chlorophylls and photoprotection. In contrast, lutein and the α-branch of the MEP pathway are not present in cyanobacteria. In this study, we genetically engineered the cyanobacterium *Synechocystis* for the missing MEP α-branch resulting in lutein accumulation. A cassette comprising four *Arabidopsis thaliana* genes coding for two lycopene cyclases (*AtLCYe* and *AtLCYb*) and two hydroxylases (*AtCYP97A* and *AtCYP97C*) was introduced into a *Synechocystis* strain that lacks the endogenous, cyanobacterial lycopene cyclase *cruA*. The resulting *synlut* strain showed wild-type growth and only moderate changes in total pigment composition under mixotrophic conditions, indicating that the *cruA* deficiency can be complemented by *Arabidopsis* lycopene cyclases leaving the endogenous β-branch intact. A combination of liquid chromatography, UV-Vis detection and mass spectrometry confirmed a low but distinct synthesis of lutein at rates of 4.8 ± 1.5 nmol per liter culture at OD_730_ (1.03 ± 0.47 mmol mol^–1^ chlorophyll). In conclusion, *synlut* provides a suitable platform to study the α-branch of the plastidic MEP pathway and other functions related to lutein in a cyanobacterial host system.

## Introduction

Carotenoids consist of unbranched hydrocarbon chains, which are terminated by ionone rings. They are found in photosynthetic organisms, as well as in some non-photosynthetic bacteria and fungi (Goodwin, 1980). They are directly involved in photosynthesis and photoprotection, and serve as precursors for the synthesis of certain hormones. Moreover, large-scale industrial production of carotenoids for use as food colorants, additives and antioxidants is of considerable commercial interest (Lau et al., 2015).

**FIGURE 1:**
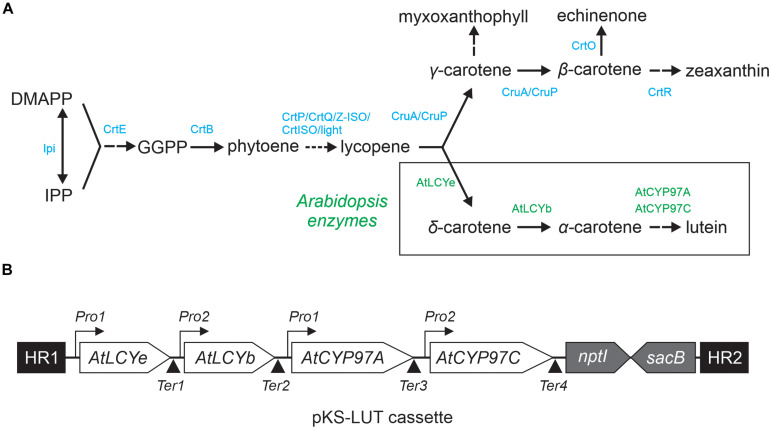
Engineered lutein synthesis pathway in *Synechocystis.* **(A)** Endogenous carotenoid and engineered lutein synthesis pathways in *Synechocystis*. Two plant lycopene cyclases (AtLCYe and AtLCYb) and two hydroxylases (AtCYP97A and AtCYP97C) create a novel synthesis pathway that diverges from the CruA/CruP branch and converts lycopene to lutein. The endogenous *Synechocystis* and introduced *Arabidopsis* enzymes necessary for lutein production are marked in blue and green, respectively. Abbreviations: IPP: isopentenyl diphosphate, DMAPP: dimethylallyl diphosphate, GGPP: geranylgeranyl diphosphate, CrtE: geranylgeranyl diphosphate synthase, CtrB: phytoene synthase, CrtP: phytoene desaturase, CtrQ: zeta carotene desaturase, Z-ISO: 15-*cis*-ζ-carotene isomerase, CrtISO: *cis*-to-*trans* carotenoid isomerase, light: photoisomerization, CruA/CruP: CruA/CruP-type of lycopene cyclases (CruA: β*-*cyclase activity, CruP: not proven β*-*cyclase activity), CrtO: β-carotene ketolase, and CrtR: β-carotene hydroxylase. A more detailed overview of the carotenoid biosynthesis pathway in cyanobacteria is given elsewhere (Domonkos et al., 2013; Sugiyama and Takaichi, 2020). **(B)** Schematic representation of the four-gene pKS-LUT construct used for lutein synthesis in *Synechocystis*. The construct is designed to integrate into a neutral site (*slr0168*) in the *Synechocystis* genome, via double homologous recombination mediated by the two flanking sequences HR1 and HR2. *AtLCYe*: ε-cyclase, *AtLCYb*: β-cyclase, *AtCYP97A*: β-hydroxylase, and *AtCYP97C*: ε-hydroxylase. Each *Arabidopsis* gene is under the control of either the *Pro1* (*psbA2, slr1311* derived) or the *Pro2* (*rbcL, slr0009* derived) promoter. After each open reading frame a specific terminator was set. *Ter1* and *Ter2* (*slr0012* derived), *Ter3* (*sll1389* derived) and *Ter4* (*slr1311* derived). See the NCBI BioProject: PRJNA731655. The pKS-LUT vector includes a double selection cassette that confers kanamycin resistance (*nptI*) and sucrose sensitivity (*sacB*).

Carotenoids are derived from the isoprenoid biosynthetic pathways. The mevalonate (MEV) pathway takes place in the cytosol of plant cells, while the methyl-erythritol phosphate (MEP) pathway is localized in plastids (Lichtenthaler et al., 1997). The first step in the carotenoid pathway *per se* is the synthesis of phytoene, which is then converted *via* several desaturation steps into lycopene, the branch-point for the generation of all other carotenoids (Figure 1A; Domonkos et al., 2013; Sugiyama and Takaichi, 2020). In principle, plants and cyanobacteria share the same lycopene synthesis pathway, but they differ in the subsequent cyclization steps (Paniagua-Michel et al., 2012; Breitenbach et al., 2013). The enzymes that create the rings at the ends of the hydrocarbon chain belong to the lycopene cyclase family (LCYs). Subsequently, cytochrome P450 (CYPs) and non-heme di-iron (HYD) enzymes are required for xanthophyll generation. In cyanobacteria, the MEP pathway is the sole pathway available for carotenoid formation (Pattanaik and Lindberg, 2015). Cyanobacteria produce mainly β-carotene, zeaxanthin and the cyanobacterium-specific xanthophylls myxol glycosides (hereafter referred to as myxoxanthophyll) and echinenone (Takaichi et al., 2001). In 2008, a new additional carotenoid – synechoxanthin - was found in *Synechococcus* and *Synechocystis* (Graham et al., 2008; Maresca et al., 2008). However, *Synechocystis* lack the plant α-branch of the MEP pathway and the xanthophylls lutein, violaxanthin and neoxanthin (Takaichi et al., 2012; Menin et al., 2019). A more detailed overview of the carotenoid biosynthesis pathway in cyanobacteria is given elsewhere (Domonkos et al., 2013; Sugiyama and Takaichi, 2020).

Lutein is the most abundant xanthophyll in plants, and is essential for stabilization of the light-harvesting complexes (LHC), transfer of excitation energy to chlorophylls and photoprotection of photosystem II (PSII) (Jahns and Holzwarth, 2012). Lutein is also employed as a food colorant and its antioxidant properties have been investigated in several studies for their therapeutic potential in cardiovascular disease (Chopra and Thurnham, 1999), atherosclerosis (Dwyer et al., 2001) and age-related macular degeneration (Seddon et al., 1994). Marigold flowers are currently the major commercial source of lutein. However, recent studies have shown that microalgae have the potential to increase lutein productivity significantly, because they outperform higher plants in terms of growth rates and do not require cropland for their cultivation (Lin et al., 2015; Sun et al., 2016).

In this work, the cyanobacterium *Synechocystis* sp. PCC 6803 (*Synechocystis*) was genetically engineered for the missing MEP α-branch and finally the production of the plant-specific xanthophyll lutein. Cao and co-workers have already shown that *Synechocystis* cells can be modified to produce lutein by interruption of the echinenone synthesis pathway and introduction of the two *Arabidopsis* enzymes lycopene cyclase LCYe (AtLUT2) and hydroxylase CYP97C (AtLUT1) (Cao et al., 2020). Here we describe a different approach, with the aim of introducing the entire MEP α-branch of higher plants into *Synechocystis* in the presence of an intact β-branch to have minimal impact on native carotenoid composition and growth performance. We demonstrate in the present study that, following the introduction of four enzymes from higher plants, *Synechocystis* is able to produce detectable amounts of lutein without dramatically altering the endogenous carotenoid composition. Such strain with ideally more enhanced lutein levels may provide a suitable cellular chassis for studying lutein-associated processes, such as the assembly of plant-type LHCs and other carotenoid-binding proteins.

## Materials and Methods

### Growth Conditions

*Escherichia coli* strains DH5α and TOP10 were grown at 37°C in lysogeny broth (LB) medium under continuous shaking at 225 rpm. The pAC-LYC (Cunningham et al., 1994) and the pAC-LYC/pKS-LUT strains were grown at 30°C in LB medium supplemented with 34 μg mL^–1^ chloramphenicol and 100 μg mL^–1^ kanamycin (pAC-LYC/pKS-LUT only).

A glucose-tolerant strain of *Synechocystis* (GT, H. Pakrasi, Department of Biology, Washington University, St. Louis) was chosen as the WT control. Unless otherwise stated, all strains were grown in blue-green-11 (BG-11) medium containing 5 mM glucose at 23°C under continuous illumination at 30 μmol photons m^–2^ s^–1^ (Rippka et al., 1979). Solid medium contained BG11 supplemented with 1.5% agar (w/v), 0.3% (w/v) sodium thiosulfate, and was buffered with 10 mM TES-KOH (pH 8.0). Cell growth was monitored with a UV-VIS spectrophotometer by recording the optical density (OD) at 730 nm. In general, cultures used for pigment extraction were grown in triplicate at 23°C under 30 μmol photons m^–2^ s^–1^, and adjusted to an OD of 0.8 at 730 nm before extraction. Cultures for lutein quantification were grown at 30°C under 100 μmol photons m^–2^ s^–1^ to a higher OD of 1.5 at 730 nm to increase lutein amounts.

### Vector Generation

For lutein synthesis in *Synechocystis*, a plasmid carrying four *Arabidopsis* genes (*AtLCYe*: ε-cyclase, AT5G57030; *AtLCYb*: β-cyclase, AT5G57030; *AtCYP97A*: β-hydroxylase, AT1G31800; *AtCYP97C*: ε-hydroxylase, AT3G53130), together with the homologous flanking regions (HR1, HR2), two different promoters (*Pro1, psbA2 derived* and *Pro2, rbcL derived*), four different terminators (*Ter1* and *Ter2*, *slr0012* derived, *Ter3*, *sll1389* derived and *Ter4*, *slr1311* derived) and the double selection cassette (kanamycin resistance [*nptI*], sucrose sensitivity [*sacB*]) was generated (Figure 1B). Gateway cloning was used for its assembly (Engler et al., 2009). The nucleotide sequences of the inserted *Arabidopsis thaliana* genes were adapted for *Synechocystis* (in terms of codon usage) with the aid of the OptimumGene-Codon Optimization tool^1^ (Genscript, Piscataway, NJ, United States). The genes were synthesized without the predicted chloroplast-transit peptide sequences (Emanuelsson et al., 1999) and placed under the control of native promoters (*Pro1, psbA2 derived* for *LCYe* and *CYP97A3*, *Pro2, rbcL derived* for *LCYb* and *CYP97C1*) (Mohamed et al., 1993; Kelly et al., 2009) and terminator sequences. The genes were cloned into the *Eco*RV sites of the cloning vector pUC57. The construct was designed to integrate into the neutral region *slr0168* (Kunert et al., 2000). The two 600-bp flanking regions were amplified from *Synechocystis* genomic DNA. The double selection cassette was amplified from pRL250 and placed downstream of the genes.

For the generation of the *cruA* insertion cassette, two 500-bp flanking regions were amplified from genomic DNA, so as to eliminate the complete *sll0147* gene from the *Synechocystis* genome. The spectinomycin resistance gene was amplified from plasmid pICH30971 and placed between the flanking regions using the Golden Gate cloning system (Engler et al., 2009). The primers used are listed in the Supplementary Table 3 and the vector sequence is given in the NCBI BioProject repository: PRJNA731655.

### Color Complementation Experiment in *E. coli*

For functional analysis of the lutein synthesis genes, pKS-LUT was introduced into pAC-LYC-containing *E. coli* cells that produce lycopene. These cells were first rendered competent and then transformed with the pKS-LUT vector.

### Generation of Transgenic *Synechocystis*

The glucose-tolerant WT *Synechocystis* strain (see above) was transformed with the plasmid pKS-LUT. Subsequently, segregated transformants were employed to disrupt *cruA* which resulted in the *synlut* strain. The strain Δ*cruA* was created by a deletion of the *cruA* gene in the same glucose tolerant wild-type strain. Transformation of *Synechocystis* wild type and mutant strains was performed as described (Williams, 1988). Briefly, *Synechocystis* cells in exponential growth phase were harvested and resuspended in BG11 to a density of 10^9^ cells mL^–1^. Plasmid DNA (2 μg) was added to the suspension and then incubated for 6 h at 25°C under illumination and (during the last 3 h) agitation. To allow these cells to recover, they were transferred to fresh BG11 and incubated at 28°C overnight in the dark. Then, the cells were harvested, and plated on BG11 agar plates supplemented with the appropriate antibiotic. To achieve complete segregation of the transformed from the starting strain, the antibiotic concentration was steadily increased (up to 100 μg mL^–1^ spectinomycin for Δ*cruA* and additionally 100 μg mL^–1^ kanamycin for *synlut*). Genomic DNA was isolated by the xanthogenate-SDS method (Tillett and Neilan, 2000). To verify correct integration of the constructs into the chromosome, diagnostic PCRs were performed with specific primers (Supplementary Table 3) and genomic DNA from the mutant strains as template.

### Whole-Genome Re-Sequencing Analyses

Genomic DNA for whole-genome re-sequencing was extracted from 5 mL of late exponential-phase cultures grown mixotrophically in BG11 supplemented with 5 mM glucose at 23°C under 30 μmol photons m^–2^ s^–1^. DNA was extracted using the xanthogenate method (Tillett and Neilan, 2000), and DNA integrity was confirmed by agarose gel electrophoresis and subsequent ethidium bromide staining. Illumina MiSeq genome re-sequencing (5 × 10^6^, 2 × 250 bp paired-end reads) was performed by the in-house sequencing service at the LMU Biocenter. Reads were mapped onto either the *Synechocystis* sp. PCC6803 WT reference genome (Genbank ID BA000022) or the transformed plasmid, using CLC Genomics Workbench (Qiagen, Venlo, Netherlands); coverage statistics, sliding-window average coverage scores, and consensus sequences were obtained using Geneious (Geneious Prime 2020.1.1)^2^.

### Reverse Transcription PCR

Cultures of WT and mutant strains were harvested in the exponential growth phase for RNA isolation. Total RNA was extracted using the Trizol reagent (Thermo Fisher Scientific, Waltham, MA, United States) according to manufacturer’s instructions. Total RNA (1 μg) was reverse-transcribed to cDNA with the iScript cDNA synthesis kit (Bio-Rad, Hercules, CA, United States). For normalization of transcript levels, the 16S rRNA abundance was used as control. Transcription of the heterologous expressed genes was checked with specific oligonucleotides, which are listed in the Supplementary Table 3.

### Northern Blot Analysis

Northern blot analysis was performed using 5 μg of total RNA. The RNA was fractioned by denaturing agarose gel (1.2%) electrophoresis and blotted onto a nylon membrane (Hybond N+; GE Healthcare, Freiburg, Germany) (Sambrook and Russel, 2001). Transcripts were detected with radioactive ^32^P[dCTP]-labeled probes, which were generated by the random primer method. The probes were amplified from *Arabidopsis* genomic DNA using the primers listed in the Supplementary Table 3. Signals were detected with a Typhoon scanner (GE Healthcare, Freiburg, Germany).

### Pigment Extractions and Analyses

Samples of *Synechocystis* (1 mL) and *E. coli* (10 mL) cells used for absorption-spectrum analysis were grown to OD 1 at 730 nm (*Synechocystis*) or 600 nm (*E. coli*), and harvested by centrifugation. Total non-polar pigments were extracted using 1 mL of 100% methanol. The samples were incubated on ice for 30 min in the dark, with intermediate vortexing (600 rpm). The extraction procedure was repeated until the cell pellets were colorless in the case of *E. coli*, and bright blue in the case of *Synechocystis* (owing to the phycocyanobilin remaining in the precipitated protein fraction). The cell debris was removed by centrifugation and the supernatants were measured in a spectrophotometer (350–800 nm). To quantify the *Synechocystis* and *E. coli* pigments, the samples were extracted as described above using 100% acetone The samples were directly subjected to high-performance liquid chromatography analysis, as described by Faber and co-workers (Farber et al., 1997). Pigment peaks of the HPLC runs were annotated using appropriate standards and quantified by their specific extinction coefficient. The standards were purified from *Synechocystis* extracts by thin-layer chromatography and identified on the basis of their spectra. The HPLC analyses revealed a low-intensity lutein peak with a retention time close to that of zeaxanthin.

To improve lutein detectability and the difference in retention time between the two pigments for absolute quantification, a new chromatographic strategy was employed. First, cultures were grown at higher temperature (30°C) and under higher light intensities (100 μmol photons m^–2^ s^–1^) to an OD of 1.5 at 730 nm. Secondly, larger culture volumes (50 mL) were harvested. Thirdly, the extraction, separation, and detection methods were specifically tailored to lutein. In brief, 1.5 mL of 100% methanol was used for overnight extraction in the dark at 4°C, with continuous mild shaking (300 rpm). Cell debris was removed by centrifugation and the supernatant was fractionated on a home-made C_18_ column (C_18_ reversed-phase silica gel, Merck, Darmstadt, Germany), pre-equilibrated with mixture 1 (acetone/methanol, 40/60 v/v). Elution was performed under gravity flow using mixture 1. The fractions were dried *in vacuo* on a sample concentrator, and stored at −80°C until analysis. For absolute quantification of lutein, the pellet was dissolved in 100 μL of 100% methanol. The analyses were performed on a Dionex Ultimate 3000 UHPLC including a diode array detector (DAD) (Thermo Fisher Scientific, Waltham, United States). Furthermore, a timsTOF (Bruker Daltonics, Bremen, Germany) was used to verify the mass spectrum, fragments, and isotopic pattern. Samples (10 μL) were injected and separated at a flow rate of 500 μL min^–1^ on a C_30_ reversed-phase column (Acclaim C_30_, 3 μm, 2.1 × 150 mm, Thermo Fisher Scientific, Waltham, United States) at 15°C. The solvents used were (A) acetonitrile and (B) a mixture of methanol and ethyl acetate (50/50 v/v), both containing 0.1% formic acid. The gradient started with 14.5% B followed by a ramp to 34.5% B within 15 min. The latter was maintained for 10 min, before returning to 14.5% B with additional 5 min of re-equilibration. Lutein was identified using a commercial standard (Extrasynthese, Genay Cedex, France), by retention time, specific *m/z* values, MS/MS fragmentation and the true isotopic pattern, as well as by the full absorption spectrum of the DAD. For absolute quantification, the lutein standard was spiked into *Synechocystis* WT sample extracts to create a calibration curve within the natural background. Three different concentrations (100 pM, 1 nM, 10 nM, each 4 replicates) were added to aliquots of the same *Synechocystis* wild-type culture. A non-spiked *Synechocystis* wild-type was used to perform baseline subtraction. Using *Synechocystis* as background for quantification kept potential matrix effects equal between calibrant and sample. Lutein was quantified using the DAD signal at 450 nm. Mass spectrometry was used for lutein identification. Data were acquired by otofControl 4.0 in positive MS mode from the 50–1300 *m/z* mass range, and evaluated using DataAnalysis 5.0 and MetaboScape 4.0. All software tools were provided by Bruker. In general, three independent biological replicates, grown in parallel in separate culture batches, were used for each analysis.

## Results

### The Lutein Synthesis Construct

Lutein production under photoautotrophic conditions in *Synechocystis* requires that endogenous synthesis pathways for essential carotenoids are not significantly impaired. Thus, a four-gene construct was designed (Figure 1B) and assembled into the vector pKS-LUT (NCBI BioProject: PRJNA731655) which provides for homologous recombination into the neutral genomic locus *slr0168* (Kunert et al., 2000). The step of β-cyclization and β*-*hydroxylation could be achieved by endogenous CruA and CrtR activity. However, it is known that LCYb is important to sustain LCYe mono-cyclase activity (Bai et al., 2009) and ε*-* and β-hydroxylases require synergistic interaction to form lutein (Quinlan et al., 2012). Consequently, *Arabidopsis* genes coding for the ε-cyclase LCYe (At5G57030), β-cyclase LCYb (At3G10230), ε-hydroxylase CYP97C1 (At1G31800), and β-hydroxylase CYP97A3 (At3G53130) were optimized with respect to their codon-usage frequency for use in *Synechocystis*. For efficient transcription, either *psbA2* or *rbcL* promoter sequences were placed upstream of each of the *Arabidopsis* genes, omitting ChloroP-predicted (Emanuelsson et al., 1999) transit peptide sequences (Figure 1B). Important promoter elements – such as the σ^70^ motif, ribosome-binding and transcription activation sites, and the *cis*-element in the *P_*psbA*2_* promoter that is responsible for light regulation (Mohamed et al., 1993; Kelly et al., 2009) – were left intact. Finally, the various building blocks were assembled into pKS-LUT using the Golden Gate Shuffling Cloning system, which permits seamless ligation of up to 10 fragments in a single reaction (Engler et al., 2009).

### Lutein Synthesis in *E. coli*

**FIGURE 2:**
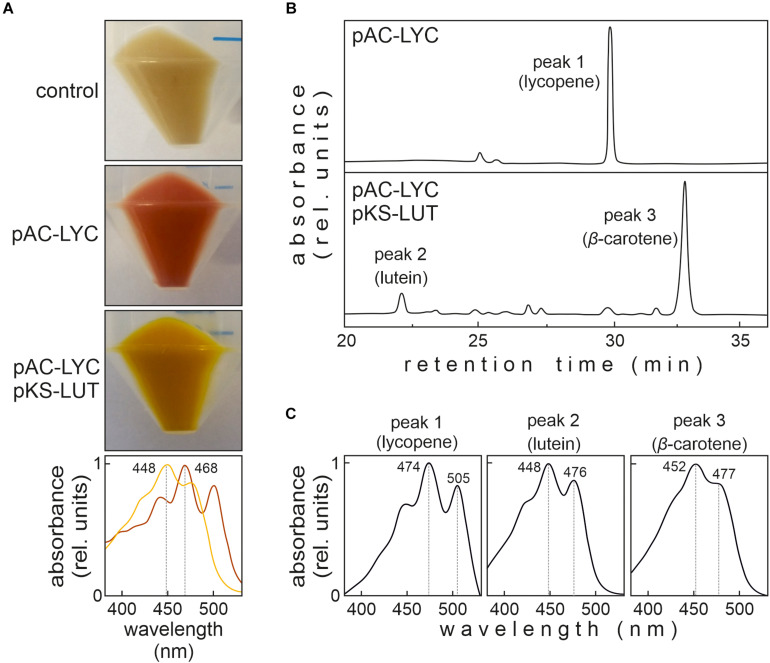
Color complementation assay in *Escherichia coli.* **(A)** *E. coli* cells transformed with either pAC-LYC or pAC-LYC/pKS-LUT. Bottom panel: Absorption spectrum of total pigments extracted from pAC-LYC (red line) or pAC-LYC/pKS-LUT (orange line). Absorption maxima are indicated. **(B)** HPLC analyses of extracts of *E. coli* cells transformed with pAC-LYC or pAC-LYC/pKS-LUT. **(C)** Reference spectra for lycopene, lutein and β-carotene. pAC-LYC, plasmid containing the genes for lycopene production. pKS-LUT, plasmid containing the genes to generate lutein from lycopene.

**FIGURE 3:**
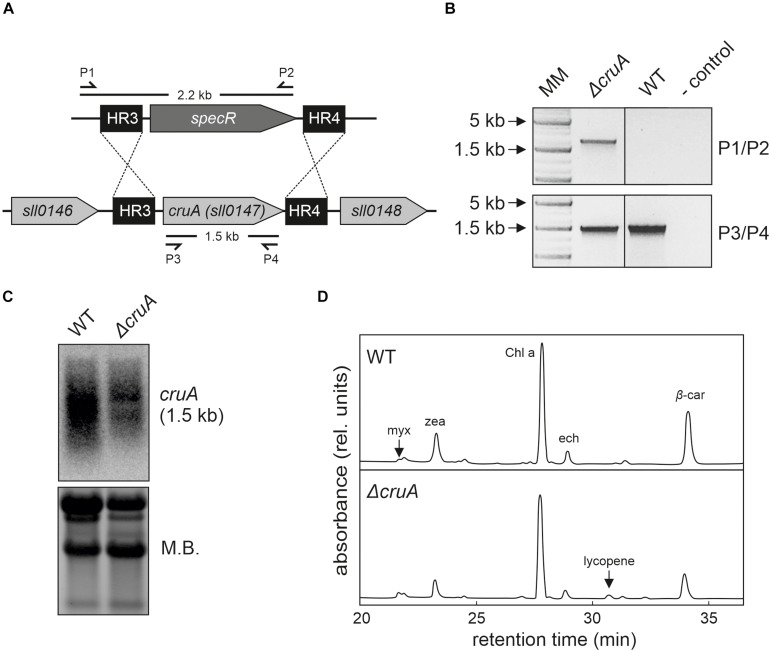
Isolation of the Δ*cruA* knockdown strain. **(A)** Design of the *cruA* insertion cassette. A spectinomycin resistance-mediating gene was inserted between two sequences (HR3 and HR4) designed to mediate the deletion of the complete *cruA* coding sequence (*sll0147*). **(B)** Characterization of the Δ*cruA* knockdown strain and the segregation status by PCR analyses. Primer-binding sites and product lengths are shown in panel **(A)**. P1–P4: primers (see Supplementary Table 3 for further information), MM: molecular marker. Full-length gel of panel **(B)** is presented in the Supplementary Figure 2A. **(C)** RNA gel-blot hybridization analyses with total RNA isolated from wild type (WT) and Δ*cruA*. After fractionation on a denaturing RNA gel and transfer to a nylon membrane, *cruA* transcripts were identified with radioactively labeled [α−^32^P]dCTP DNA probes. Equal loading was checked by staining the nylon membranes with methylene blue solution (M.B.). Full-length blots of panel **(C)** are presented in the Supplementary Figure 2B. **(D)** HPLC analysis of WT and the two Δ*cruA* mutants grown under low-light conditions (30 μmol photons m^– 2^ s^– 1^). Major pigments and lycopene (peak at retention time 30.25 min in the Δ*cruA* sample) are indicated. myx: myxoxanthophyll, zea: zeaxanthin, Chl *a*: chlorophyll *a*, ech: echinenone, and β*-*car: β-carotene.

To test whether all four enzymes are functional, pKS-LUT was transformed into a lycopene-accumulating *E. coli* strain carrying the plasmid pAC-LYC containing the genes for lycopene production (Cunningham et al., 1996). The change in the color of *E. coli* cells from red to orange was the first indication of the conversion of lycopene to other carotenoids (Figure 2A), as was confirmed by a shift in the maximum absorption of isolated total pigments from 468 nm in cells bearing pAC-LYC to 448 nm in pAC-LYC/pKS-LUT. To determine the carotenoid compositions of the pAC-LYC and pAC-LYC/pKS-LUT strains, their pigments were extracted and analyzed by high-performance liquid chromatography (HPLC). The *E. coli* pAC-LYC/pKS-LUT strain accumulated two major pigments (Figure 3B) and comparison of their retention times and absorption spectra with commercial standards proved that these were lutein and β-carotene. (Figures 3B,C). Based on HPLC quantitation, β-carotene (13.8 nmol ml^–1^) was produced at a considerably higher level than lutein (0.8 nmol ml^–1^). Together, these results demonstrate that the four enzymes encoded by the cassette present in pKS-LUT were functionally expressed and enabled the production of lutein in *E. coli.*

### Integration of the Lutein Synthesis Pathway Into *Synechocystis*

Next, the pKS-LUT cassette was stably integrated by homologous recombination into the neutral site *slr0168* in the genome of the wild-type (WT) *Synechocystis* strain. However, no lutein was detected in HPLC analyses of the pigments isolated from the resulting strain (data not shown). This finding suggested that either one or more of the introduced *Arabidopsis* enzymes were not sufficiently expressed, their activity too low, or that the endogenous carotenoid pathways effectively outcompeted for the common substrate lycopene. To test this hypothesis, the *cruA* gene that codes for the endogenous *Synechocystis* β-cyclase (Maresca et al., 2007; Xiong et al., 2017) was disrupted in order to redirect the synthesis pathway away from β-carotene and zeaxanthin toward lutein.

### Disruption of the Endogenous *Synechocystis* β-Cyclase

First, we investigated the effects of β-cyclase disruption in the absence of the engineered lutein synthesis pathway. To this end, we tried to generate a *cruA* knockout mutant strain (Δ*cruA*) in the WT background by replacing the *sll0147* coding sequence with a spectinomycin resistance-mediating gene (Figure 3A). Successful integration was verified by PCR in the Δ*cruA* strain (Figure 3B). However, even after selection on high concentrations of spectinomycin (up to 100 μg mL^–1^), the insertion cassettes of the strain did not segregate completely (Figure 3B). To determine the level of transcription of the β-cyclase gene, RNA gel-blot hybridization analyses were carried out on samples of total RNA isolated from WT and Δ*cruA* (Figure 3C). Since the expression of *sll0147* was indeed significantly reduced in the Δ*cruA* mutant, it was used as *cruA* knockdown in subsequent analyses.

**FIGURE 4:**
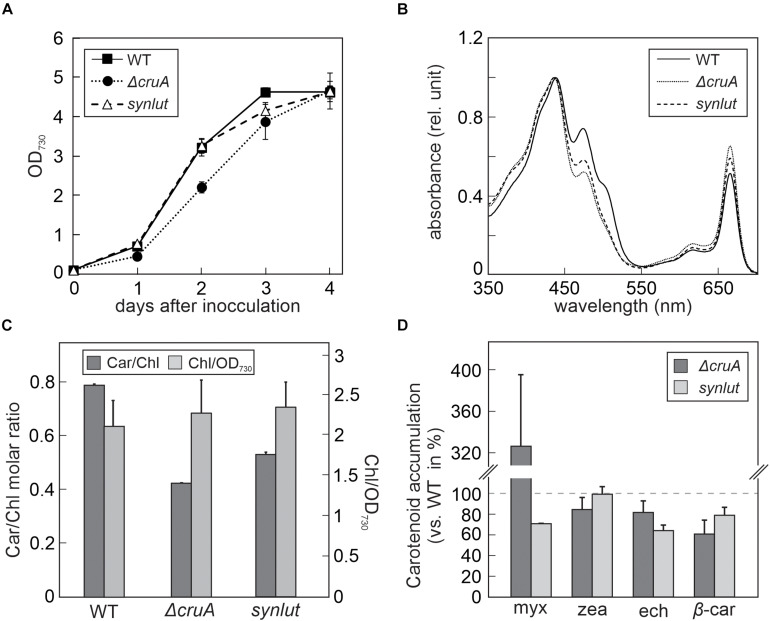
Growth rates and pigment composition of Δ*cruA* and *synlut*. **(A)** Growth curves of Δ*cruA* and *synlut* in comparison to *Synechocystis* wild-type (WT) cells. Cultures were grown mixotrophically in blue-green-11 (BG-11) medium containing 5 mM glucose at 23°C under continuous illumination at 30 μmol photons m^– 2^ s^– 1^. The optical density (OD) of *Synechocystis* cultures was photometrically determined daily at a wavelength of 730 nm. Means and standard deviations of three biological replicates are shown. **(B)** Total absorption spectra of pigment samples from WT, Δ*cruA* and *synlut* cells. Spectra were normalized to the maximal absorption value. **(C)** Molar ratios of total carotenoids to chlorophyll *a* (Car/Chl) and chlorophyll *a* content (normalized to the amount in 1 mL of cells at OD_730nm_ = 1) in WT, Δ*cruA* and *synlut*. Values represent averages of three biological replicates and two independent experiments. Error bars indicate standard deviations. **(D)** Levels of major carotenoids in Δ*cruA* and *synlut* relative to WT. Means of three replicates are shown, and error bars represent standard deviations. Abbreviations: myx: myxoxanthophyll, zea: zeaxanthin, ech: echinenone, and β*-*car: β-carotene.

To study the effect of β-cyclase disruption on pigment composition, HPLC analyses (Figure 3D) were performed with pigments isolated from Δ*cruA* knockdown strain as described previously (Farber et al., 1997). Levels of zeaxanthin, echinenone and β*-*carotene were significantly reduced in Δ*cruA* (Figures 3D,4D). As expected, lycopene accumulated in Δ*cruA*, indicating that its conversion to β*-*carotene was less efficient when the endogenous β-cyclase activity was depressed.

### *cruA* Knockout in the Presence of the Lutein Synthesis Pathway Genes

**FIGURE 5:**
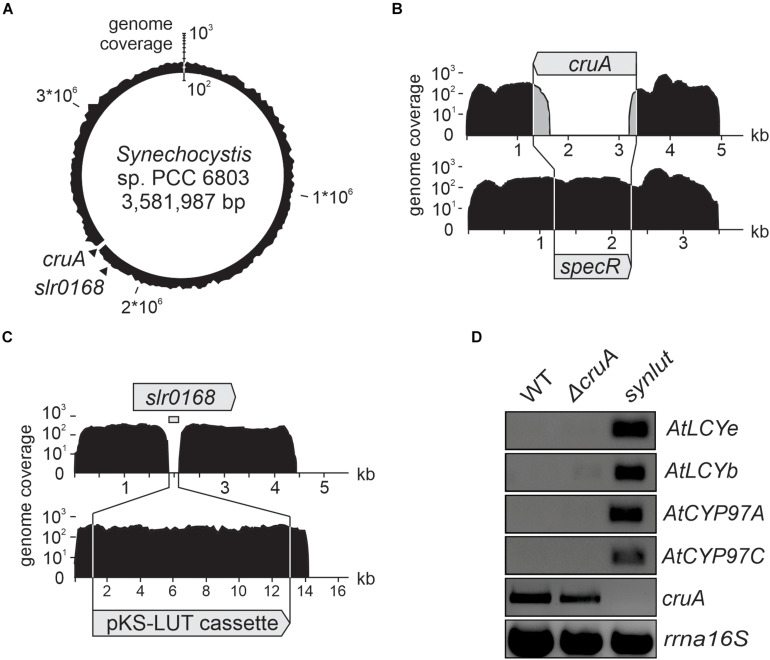
Whole-genome resequencing and expression analysis of *synlut*. **(A–C)** Schematic depiction of the density of coverage of the **(A)** *synlut* chromosome, **(B)** of the *cruA* locus in *synlut* and **(C)** of the pKS-LUT cassette in the neutral integration site *slr0168*. Sequence reads assembled in **(B)** and **(C)** were either mapped to the wild type (WT, upper panel) or the *synlut* reference genome (lower panel). Genome coverage values in **(A–C)** are shown on a logarithmic scale. Gray shading in **(B)** indicates false alignments of sequence read ends. Absolute values are listed in Table 1. **(D)** Reverse transcription-PCR analyses of RNA samples isolated from WT, Δ*cruA* and *synlut* strains. *AtLCYe*, *AtLCYb*, *AtCYP97A*, *AtCYP97C* and *rrnA16S* cDNAs were amplified over 25 cycles, and the *cruA* cDNA was subjected to 30 PCR cycles, respectively. A PCR-based segregation analysis of *synlut* is provided in the Supplementary Figure 1. Full-length gels of panel **(D)** are presented in the Supplementary Figure 2C.

In the next step, a strain with stably integrated pKS-LUT cassette was transformed with the *cruA* insertion cassette (Figure 3A) and progenies were selected on high concentrations of spectinomycin. The resulting strain was named *synlut* and subjected to whole genome re-sequencing (Figure 5). Reads were mapped to the *Synechocystis* sp. PCC6803 WT reference genome with a coverage of 249 ± 62 and an Illumina quality score (>Q20) of 93% (Figure 5A and Table 1). Interestingly, examination of the *cruA* locus (Figure 5B and Table 1) in *synlut* revealed that it had been completely lost, as indicated by the absence of corresponding sequence reads. We also verified the genetic integrity of the pKS-LUT cassette and the correct double homologous recombination event into the neutral site *slr0168* in *synlut*. As in the case of the pKS-LUT cassette, complete segregation was observed for the *cruA* knockout in the neutral region *slr0168* (Figure 3C and Table 1) of *synlut*.

**TABLE 1:** Analysis of genetically modified loci in synlut by whole-genome re-sequencing.

Locus	MV	SD	>Q20 (in%)
whole chromosome	249	62	94
*cruA (sll0147)*	0	0	0
*specR* (Δ*cruA)*	244	61	90
*slr0168* total	251	118	93
*slr0168* insertion site	0	0	0
pKS-LUT cassette	273	50	100

*Fold per-site coverage mean values (MV) and corresponding standard deviations (SD), as well as percentage of mapped reads with Illumina quality score >20 (Q20; i.e., percentage of reads with an inferred per-site base call accuracy >99%).*

*Data were obtained by mapping synlut sequencing reads to the Synechocystis sp. PCC 6803 wild-type reference genome (BA000022) using CLC Genomics Workbench.*

To determine the expression levels of the four *Arabidopsis* genes encoded by the pKS-LUT cassette, reverse transcription-PCR analyses were performed (Figure 5D). All genes were transcribed and, as expected from the whole genome re-sequencing analyses, no *cruA* transcripts could be detected in *synlut*. Moreover, the incomplete segregation of Δ*cruA* already identified in RNA gel-blot hybridization analyses (Figure 3C) was confirmed by the detection of *cruA* cDNAs upon increasing the number of PCR cycles performed.

The impact of the *cruA* knockdown and genetic modifications in *synlut* on growth performance and pigment composition was then examined in more detail (Figure 4). While *synlut* showed wild-type-like growth rate, the Δ*cruA* knockdown did not reach a comparable growth performance (Figure 4A). The doubling time of Δ*cruA* (13.9 ± 1.2 h) during the exponential growth phase was ∼ 40% longer compared to the WT (9.6 ± 0.8 h) and the restored *synlut* strain (9.6 ± 0.6 h). UV-Vis spectra of total pigments isolated from both Δ*cruA* and *synlut* revealed a lower absorption value at 460 nm compared to wild-type samples, which pointed to a lower carotenoid content in both strains (Figure 4B). Indeed, the molar ratios of total carotenoids to chlorophyll *a* in Δ*cruA* (0.424 ± 0.002) and *synlut* (0.530 ± 0.009) were significantly lower than that of the wild type (0.790 ± 0.001), although levels of chlorophyll *a* were similar in all genotypes (Figure 4C). Quantification of major carotenoids by HPLC demonstrated that the Δ*cruA* knockdown produced less zeaxanthin (85 ± 11%), echinenone (82 ± 12%) and β-carotene (61 ± 14%), but more myxoxanthophyll (326 ± 70%) relative to WT samples. In *synlut*, the zeaxanthin level (100 ± 7%) was equal to that in the Δ*cruA* strain, but amounts of myxoxanthophyll (70 ± 0.5%) and echinenone (64 ± 6%) were lower (Figure 4D). The restoration of zeaxanthin to WT levels and the increase in β-carotene to 79% (±8%) of WT in *synlut* indicated that the transgenic *Arabidopsis* cyclases of the α-branch were able to functionally replace the endogenous *Synechocystis* β-cyclase of the β-branch. Consequently, full segregation of the *cruA* knockout could be achieved in the presence of the *Arabidopsis* enzymes in *synlut* (Figure 5).

### Lutein Quantification in *synlut*

**FIGURE 6:**
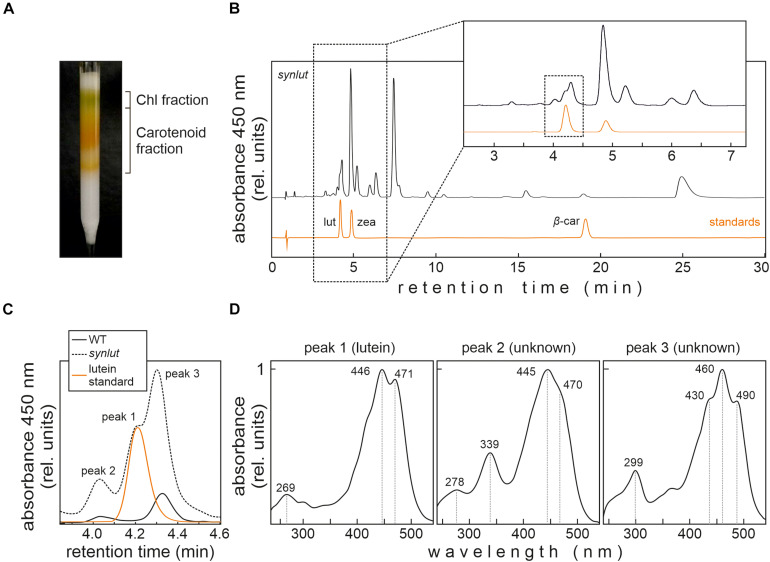
Lutein identification in *synlut*. **(A)** Preparative C18 column for extract purification, pre-fractionation and pigment enrichment prior to UHPLC-MS analysis. The two major fractions indicated were collected for further analyses. **(B)** UHPLC analysis of the *synlut* carotenoid fraction and a mixture of the indicated standards (lut, lutein; zea, zeaxanthin; β-car, β-carotene). Separation was followed by recording the absorbance at 450 nm over 30 min. An expanded view of the separation of pigments with retention times between ∼2.5 and 7.0 min is shown (highlighted by the dotted box). **(C)** Enlarged view of the interval shown by the dotted box in **(B)**, showing the lutein standard and peaks identified in *synlut* and wild-type (WT) samples. **(D)** Absorption spectra of three peaks with similar retention times to lutein identified in *synlut*.

In order to detect small amounts of lutein in *Synechocystis*, an optimized pigment extraction and carotenoid separation procedure was developed (Figure 6). First, *Synechocystis* cells were grown mixotrophically at higher light intensities (100 μmol photons m^–2^ s^–1^) to enhance the light-inducible activity of the *psbA2* promoters that drive the transgenes *AtLCYe* and *AtCYP97A*. Second, crude extracts were pre-fractionated using a home-made C18 column to reduce sample complexity and concentrate carotenoids (Figure 6A). Finally, pigments were run on a UHPLC using a C_30_ reversed-phase column optimized for the separation of carotenoids, which were detected using a DAD and mass spectrometry. This procedure enabled clear-cut separation of the isomers lutein and zeaxanthin, with a retention time difference of 0.8 min (Figure 6B). However, separation of carotenoid fractions resulted in the identification of three *synlut*-specific peaks with retention times similar to the lutein standard (Figure 6C). Examination of the absorption spectrum of peak 1, which appeared with the same retention time as the standard (4.2 min), confirmed that this peak indeed contained lutein (Figure 6D). Peak 2 and 3 showed also a carotenoid-like absorption spectrum and could represent isomeric carotenoid forms such as the *cis*-form of lutein.

**FIGURE 7:**
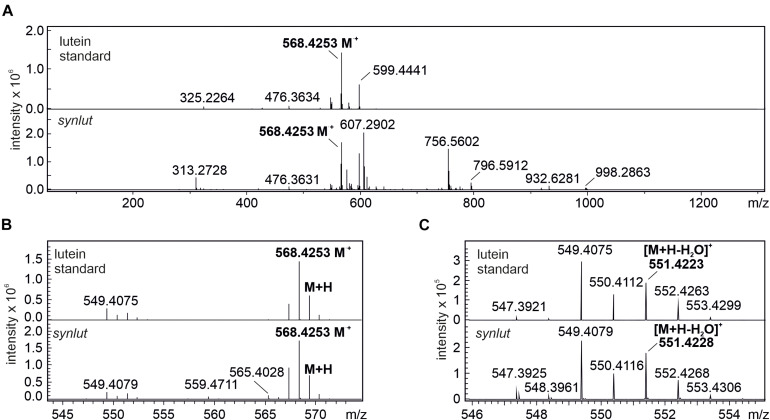
MS spectra of lutein standard and peak 1 at retention time 4.2 min. **(A)** full spectrum scan (50–1300 *m/z*) of the lutein standard and *synlut* at retention time of 4.2 min. **(B,C)** Close-up views of the mass range sections **(B)** 543–574 *m/z* and **(C)** 546–555 *m/z*. The radical ion formed by lutein (M^⋅+^, 568.4253 *m/z*) and its water loss ion ([M + H-H_2_O]^+^, 551.4228 *m/z*) are highlighted in bold letters.

Furthermore, the lutein standard and *synlut* carotenoid extracts with a retention time of 4.2 min on the C_30_ column, were examined in more detail by mass spectroscopy (Figure 7A). As was also observed for the lutein standard, *synlut* samples contained the radical (M^+^, 568.4253 *m/z*) (Figure 7B) and the characteristic water-loss ion of lutein ([M + H-H_2_O]^+^, 551.4228 *m/z*) (Figure 7C), which was also identified in an earlier study (Fu et al., 2012). Accordingly, lutein is produced by *synlut*, at rates equivalent to 4.8 ± 1.5 nmol per liter culture at OD_730_ (1.04 ± 0.47 mmol mol^–1^ chlorophyll), which corresponds to 0.5 ± 0.3% of the zeaxanthin or 0.145 ± 0.002% of the total carotenoid level in the strain.

## Discussion

In the present study, a metabolic bifurcation was successfully introduced into the carotenoid synthesis pathway of *Synechocystis*. A set of four *Arabidopsis* enzymes (Figure 1B) encoded by the stably integrated genetic cassette pKS-LUT was able to divert metabolic fluxes through the introduced MEP α-branch toward lutein. The major challenge in this work was to balance lutein production with endogenous pigment synthesis, since the newly introduced pathway must compete with endogenous reactions for the common substrate lycopene. In addition, parallel synthesis of lutein and endogenous pigments requires tight coordination between ε- and β-cyclization of the ionone rings in lycopene. These problems are clearly illustrated by our failure to produce lutein following transformation of the pKS-LUT cassette into the *Synechocystis* WT background, while the presence of the same cassette in pAC-LYC-containing *E. coli* cells led directly to lutein synthesis (Figure 3).

### Competing Pathways Lead to Moderate Lutein Levels but Unimpaired Growth

Since a *cruA* knockout mutant of *Synechococcus* sp. PCC 7002 accumulates substantial amounts of lycopene (Maresca et al., 2007; Xiong et al., 2017), we modulated the endogenous lycopene β-cyclase activity to promote lutein synthesis. However, in contrast to *Synechococcus* sp. PCC 7002 (Xiong et al., 2017), full segregation of the *cruA* knockout could not be achieved in *Synechocystis* (Figure 3). Presumably, intact copies of *cruA* had to be retained and a complete knockout may not be viable even under heterotrophic conditions, since endogenous carotenoids are required to structure the cell wall of *Synechocystis*, to organize the thylakoid membranes and to serve as a defense against reactive oxygen species (ROS) (Mohamed et al., 2005; Kusama et al., 2015). Nevertheless, in line with observations in *Synechococcus*, the *cruA* knockdown of *Synechocystis* grew more slowly and accumulated less zeaxanthin and β-carotene, but produced more lycopene (Figure 3D). Thus, these data provide further evidence that *cruA* (*sll0147*) encodes the lycopene β-cyclase in *Synechocystis*. In contrast, for the paralog CruP no β-cyclase activity could be proven (Figure 1A; Sugiyama et al., 2017; Sugiyama and Takaichi, 2020). Rather, CruP seems to be involved in ROS scavenging processes (Bradbury et al., 2012). Thus, CruP cannot complement the β-cyclase activity of CruA in *Synechocystis*. Remarkably, the transgenic *Arabidopsis* cyclases could replace the β-cyclase activity of CruA and led to full segregation of the *cruA* knockout in *synlut* (Figure 5). This finding is an important prerequisite for metabolic engineering of the critical branch point in carotenoid biosynthesis.

Interestingly, the myxoxanthophyll content in the *cruA* knockdown strain increased compared to the WT (326 ± 70%), while other carotenoid levels of the β-branch decreased (Figure 3D). The higher myxoxanthophyll level could be attributed to unbalanced fluxes at the γ-carotene branch point (Figure 1A). Since both, myxoxanthophyll and β-carotene synthesis compete for the γ-carotene pool, a reduced β-cyclase activity could lead to a diversion of fluxes toward the unaltered myxoxanthophyll synthesis pathway in *cruA*. Alternative explanations for the high myxoxanthophyll levels in *cruA* might be that other, unknown enzymes synthesize lycopene to γ-carotene without further β-carotene generation or convert lycopene directly to myxoxanthophyll as indicated by Zhang and co-workers (Zhang et al., 2015).

Lutein could only be detected in *Synechocystis* after knocking out *cruA* in the presence of the introduced MEP α-branch. Conversely, lutein could not be detected in the simultaneous presence of *cruA* and the transgenic MEP α-branch. This indicates that the low level of lutein in *synlut* is not simply due to too low expression level or low activity of the introduced enzymes. It is more likely an effect of metabolic competition for lycopene by the transgenic α*-* or the endogenous β-branch of the carotenoid biosynthesis.

### *synlut* as Cellular Chassis

Besides its economic potential, *synlut* can also serve as a photoautotrophic platform for the investigation of lutein-associated processes. Lutein is a structural component of the light-harvesting complexes (LHCs), and acts as an accessory pigment, which extends the absorption spectra of chlorophylls in land plants (Liu et al., 2004; Barros and Kühlbrandt, 2009). Lutein is required for LHC assembly and interacts with chlorophyll *a* (Croce et al., 1999) and, according to a previous study, LHCII from plants can be assembled in cyanobacteria and stabilized after exogenous pigment addition (He et al., 1999). The introduction of plant pigments into cyanobacteria could provide an opportunity to study the formation of membrane-integral LHCs in genetic and adaptive laboratory approaches by taking advantage of a prokaryotic expression system (Dann and Leister, 2017). All attempts to use *synlut* as a cellular chassis to modify pigment profiles and photosynthesis ideally require the absence of detrimental phenotypic effects. Previously, Cao et al. (2020) induced the production of lutein and other non-cyanobacterial carotenoids in *Synechocystis* by replacing the endogenous CrtO (Slr0088), encoding the β-carotene ketolase for echinenone production, with various exogenous genes. They employed the endogenous *Synechocystis* gene expression machinery to overcome problems such as suppressed expression rates and preferential utilization of certain precursor metabolites during metabolic channeling. They produced lutein in an echinenone-deficient *Synechocystis* background co-expressing only two *Arabidopsis* genes (LCYe/AtLUT2, CYP97C/AtLUT1) and demonstrated the functional collaboration of the endogenous *Synechocystis* enzymes CruA/CruP and CrtR with exogenous AtLUT1 and AtLUT2. Thus, introduction of LCYb and CYP97A is not essential for lutein synthesis in *Synechocystis* and might even lead to unwanted by-products (Figure 6C). However, the absence of echinenone in *Synechocystis* can result in suppression of the *de novo* synthesis of various proteins, and provoke singlet oxygen production by impaired thermal dissipation (Kusama et al., 2015). This might trigger severe growth effects under certain conditions. Our lutein-producing strain *synlut* shows no alterations in growth phenotype, in contrast to Δ*cruA*, in which the synthesis of native carotenoids was partially suppressed (Figure 4A). This indicates that the full spectrum of native *Synechocystis* carotenoids is required to avoid deleterious effects on growth and fitness.

### Strategies to Enhance the Lutein Content

Even though we were able to demonstrate lutein synthesis, yields were low relative to zeaxanthin in *synlut* (Figure 6). One reason for this might be that the complementation of CruA by the *Arabidopsis* β-cyclase supports both the α- and β*-*branches of the MEP pathway (Cunningham and Gantt, 2001). In fact, the *Arabidopsis* β-cyclase can produce β-carotene in *E. coli* (Figure 2), in contrast to CruA that requires chlorophyll to be functional and therefore could not synthesize β-carotene from lycopene in *E. coli* (Xiong et al., 2017). Another reason might be an imbalance between the activities of the transgenic *Arabidopsis* ε- and β-cyclases. The fact that more β-carotene than lutein was produced in pAC-LYC/pKS-LUT-bearing *E. coli* cells supports this explanation (Figure 2). Indeed, the *Arabidopsis* β-cyclase is a bicyclase, and the two β-cyclization steps for β-carotene synthesis might be more efficient than α-carotene formation, which requires two different enzymes for ε- and β-cyclization. Consequently, enhancing lycopene ε-cyclization activity and subsequent α-carotene formation might be a promising strategy for the moderate enhancement of lutein production in *synlut*.

It has been shown that a fusion protein from *Ostreococcus lucimarinus* simultaneously catalyzes formation of α- and β-carotene, and their stoichiometry could be modulated by C-terminal truncations of a light-harvesting domain in the fusion cyclase (Blatt et al., 2015). Therefore, in addition to adjusting the expression rates of the two *Arabidopsis* cyclases by means of different or inducible promoters, the use of truncated versions of the fusion cyclase from *Ostreococcus lucimarinus* or lycopene ε-cyclases from other organisms with higher activity (Zhang et al., 2019) presents further options for improving lutein production in *Synechocystis*. The *synlut* strain also represents a good starting point for the application of established strategies in metabolic engineering (Lin and Pakrasi, 2019) with a view to obtaining economically viable titers of lutein. These include engineering of the carotenoid precursor pathway (Wang et al., 2014; Pattanaik and Lindberg, 2015), downregulation of alternative pigment synthesis routes (Cao et al., 2020) and adaptive laboratory evolution approaches (Sun et al., 2016).

## Data Availability Statement

The datasets presented in this study can be found in online repositories. The names of the repository/repositories and accession number can be found below: https://www.ncbi.nlm.nih.gov/sra/PRJNA731655, accession number: PRJNA731655.

## Author Contributions

ML, EV, DL, and TR conceptualized the project. ML, EV, AT, DL, and TR wrote the manuscript. ML, EV, AT, PJ, MD, LR, and AA performed the experiments. ML and TR made the figures. All authors discussed the results and approved the manuscript.

## Conflict of Interest

The authors declare that the research was conducted in the absence of any commercial or financial relationships that could be construed as a potential conflict of interest.

## Footnotes

1 www.genscript.com/codon-opt.html

2 https://www.geneious.com
