# Supplementary material for: Introduction of the Carotenoid Biosynthesis α-Branch Into Synechocystis sp. PCC 6803 for Lutein Production
Source: Front Plant Sci. 2021 Jul 6;12:699424. doi: 10.3389/fpls.2021.699424 (PMC8291087; doi:10.3389/fpls.2021.699424)
Supplement: Supplementary file 1 [file Data_Sheet_1.PDF]

SUPPLEMENTARY TABLE S1 | Strains of this study. Charactetistics, used marker and source. SmR: spectinomycin resistance, KanR: kanamycin resistance, SucR: sucrose resistance.

Strain	Characteristics	Marker	Source
WT	Wild type *Synechocystis*, glucose tolerant		H. Pakrasi
ΔcruA	Spectinomycin cassette replacing sll0147 in *Synechocystis* wt	SmR	this study
synlut	Spectinomycin cassette replacing sll0147, into *synlut* mutant strain	KanR, SucR, SmR	this study

SUPPLEMENTARY TABLE S2 | Plasmids of this study. Charactetistics, used marker and source. AmpR: ampicillin resistance, SmR: spectinomycin resistance, KanR: kanamycin resistance, SucR: sucrose resistance.

Plasmid name	Characteristics	Marker	Source
pGEM Teasy	Backbone for many plasmids	AmpR	Promega
pRL250	*nptI-sacB*, double selection cassette	KanR, SucR	P. Wolk
pKS	pGEM Teasy with nptI-sacB from pRL250	KanR, SucR	this study
pFS-us	pGEM Teasy with upstream region of *slr0168*	AmpR	this study
pFS-ds	pGEM Teasy with downstream region of *slr0168*	AmpR	this study
pPpsbAII	pGEM Teasy with *psbAII* promoter from *Synechocystis*	AmpR	this study
pPrbcL	pGEM Teasy with *rbcL* promoter from *Synechocystis*	AmpR	this study
pUC57-LCYe_opt	Codon optimized *LCYe*	AmpR	GenScript
pUC57-LCYb_opt	Codon optimized *LCYb*	AmpR	GenScript
pUC57-CYP97A_opt	Codon optimized *CYP97A3*	AmpR	GenScript
pUC57-CYP97C_opt	Codon optimized *CYP97C1*	AmpR	GenScript
pKS-LUT	pICH30791mod with *LCYe*, *LCYb*, *CYP97A*3 and *CYP97C*1 optimized sequences, with *psbAII* and *rbcL* promoters, *nptI-sacB* selection marker and *slr0168* flanking regions	SmR, KanR, SucR	this study

psll0147-up	pGEM Teasy with upstream Flanking Sequence of *sll0147*	AmpR	this study
psll0147-ds	pGEM Teasy with downstream Flanking sequence of *sll0147*	AmpR	this study
pICH30791mod	Destination vector for Golden Gate cloning. From pICH30791, with modified BsaI sites	SmR	this study
pICH30791	Entry vector for Golden Gate, used for Spectinomycin amplification	SmR	E. Weber
pSpec	pGEM+Spec	AmpR, SmR	this study
p*Δ*cruA	pICH30791mod with Spectinomycin and flanking sequences of *sll0147*	SmR	this study

SUPPLEMENTARY TABLE S3 | Primer of this study. Sequence and purpose. SmR: spectinomycin resistance, Fw, F: forward, Rv, R: reverse.

Primer name	Sequence	Purpose
**pICH30791**		
pAssembly Fw	TTTAGGTAGAGACCCTG GCA CGA CAG GTT TCC CG	Amplification of *lacZ* gene in
pAssembly Rv	TTTAAGCAGAGACCGTCTAA GAA ACC ATT ATT ATC AT	pICH30791 with BsaI sites
**pKS-LUT**		
FS us-Fw	TTTGGTCTCTAGGTTGCGAGTTCAAACTCGATAAC	Amplification of 600 bp of *slr0168* for
FS us-Rv	TTTGGTCTCCAGTAAAATCGCTCCCTCCGTGCCT	upstream flanking region
FS ds-Fw	TTTGGTCTCTCAAGCTCAAAGGGGACGAAGCCGCAG	Amplification of 600 bp of *slr0168* for
FS ds-Rv	TTTGGTCTCTAAGCATCTGCCAAAGCTGCTTCTT	
P*psbAII*-1 Fw	TTTGGTCTCCTACTTTACAAAGCTTTACAAAACT	Amplification of 100 bp of *Synechocystis*
P*psbAII*-1 Rv	TTTGGTCTCGCATTTGGTTATAATTCCTTATGTAT	*psbAII* promoter between FS us and LCYe
P*psbAII*-2 Fw	TTTGGTCTCATGAATTACAAAGCTTTACAAAACT	Amplification of 100 bp of *Synechocystis* *psbAII* promoter for CYP97A
P*rbcL*-1 Fw	TTTGGTCTCCGGTAAAATAATAACTGTCTCTGGG	Amplification of 100 bp of *Synechocystis*
P*rbcL*-1 Rv	TTTGGTCTCGCATTCTAGGTCAGTCCTCCATAA	*rbcL* promoter in front of LCYb
P*rbcL*-2 Fw	TTTGGTCTCTATCAAAATAATAACTGTCTCTGGG	Amplification of 100 bp of *Synechocystis* *rbcL* promoter in front of CYP97C
Selection cassette Fw	TTTGGTCTCACGTTGGAATTCGATTGATCCGTCGAC	Amplification of *nptI-sacB* from pRL250
Selection cassette Rv	TTTGGTCTCCCATACTTTAGGCCCGTAGTCTGCA	

ΔcruA		
sll0147 us Fw	TTTGGTCTCTAGGTTTAAAGAATGAATTGGGGTG	Amplification of 500 bp upstream of
sll0147 us Rv	TTTGGTCTCCAGTACAAAATTGCCACTGAAATCA	*cruA* gene in *Synechocystis*
sll0147 ds Fw	TTTGGTCTCTCAAGTAAATTAGGGCGACATTAAG	Amplification of 500 bp upstream of
sll0147 ds Rv	TTTGGTCTCTAAGCTGGGTCGCCTTTCTGCACCT	*cruA* gene in *Synechocystis*
Spec Fw	TTTGGTCTCCTACTCCCTGATAAATGCTTCAATAA	Amplification of SmR gene from pIC30791 (Primer P5, Fig.S3)
Spec Rv	TTTGGTCTCTCTTGTTATTTGCCGACTACCTTGG	Amplification of SmR gene from pIC30791 (Primer P2, Fig.3, Fig.S3)
cruA gene Fw	ACTGCTCTACTGTGAAGTCCCCACC	Genotyping of *ΔcruA* for complete segregation (Primer P3, Fig.3, Fig.S3)
cruA gene Rv	GCAGGTCATACTGCAAAGCTGTGTG	Genotyping of ΔcruA for complete segregation (Primer P4, Fig.3, Fig.S3)
sll0146 Fw	GCTAATGTTATTAGCTTTAACC	Genotyping of *ΔcruA* for correct integration, with Spec Rv (Primer P1, Fig.3)
synlut		
LCYe Rv	TACCAAAAGCAACAAAAAACCCGCG	Genotyping of *synlut*, with FS us-Fw
LCYb Fw	ATGGTTAGCAGCGTTGTGAGTG	Genotyping of *synlut*
LCYb Rv	CACGGTCTTGCACCAGGTTG	
CYP97C Fw	GGGTGAGTCCCGATTGGTTGA	Genotyping of *synlut*
CYP97C Rv	AACGTCAGTCAAGCAAAAAGCAC	
sacB Fw	AGCATATCATGGCGTGTAATATGGG	Genotyping of *synlut*, with FS ds-Rv
**RT-PCR**		
LCYe F	CCGATTTGCCCTTTACCAATAAC	Reverse transcription PCR of LCYe
LCYe R	ACTTCAACTTCCACACCATAGG	
LCYb F	TTATGGACTGGCGGGATAAAC	Reverse transcription PCR of LCYb
LCYb R	CGTTCAATGGGCCACAAATC	
CYP97A3 2 F	GGTGATGACGTGAGCTCTAAAC	Reverse transcription PCR of CYP97A3
CYP97A3 2 R	AATGGGAACGCTGGGAATATC	
CYP97C1 2 F	CTTGTTAGGCGGTGCTTTATTT	Reverse transcription PCR of CYP97C1
CYP97C1 2 R	CCTTCGCGTTCCACAATTTC	
RNA16s F	TAGGGTAGCGAAAGGGATTAGA	Reverse transcription PCR of RNA16s
RNA16s R	GCAGTATTCTGACCTGCGATTA	
CruaA F	CTCCACACTCCCACTGTATTAAA	Reverse transcription PCR of cruA
CruaA R	CCAGATTACAGCGACGATACTC	

**Northern Blot**		
LCYe F	CCAGTGGTGGTGGGAGTAGT	Norther blot analysis of LCYe
LCYe R	GCCCGACCAATGGTAATGGG	
LCYb F	ATGGTTAGCAGCGTTGTGAGTG	Norther blot analysis of LCYb
LCYb R	TTGCGTTCTTTCAGTTCGGG	
CYP97A3 F	ATGGTTTTTAGCAGCAGTAGCAATG	Norther blot analysis of CYP97A3
CYP97A3 R	TTTTGGTGCAAAGCGGGCAC	
CYP97C1 F	GGGTGAGTCCCGATTGGTTGA	Norther blot analysis of CYP97C1
CYP97C1 R	CGAATCAAGGTCACAGCTTT	
cruA F	GGCCGAATGAATCGGGAATGGAATA	Norther blot analysis of LCYe
cruA R	TCCCCCTGCTTGCTGGAGTTTA	
cruA probe Fw	GGCCGAATGAATCGGGAATGGAATA	Northern blot analysis of *cruA- sll0147*
cruA probe Rv	TCCCCCTGCTTGCTGGAGTTTA	

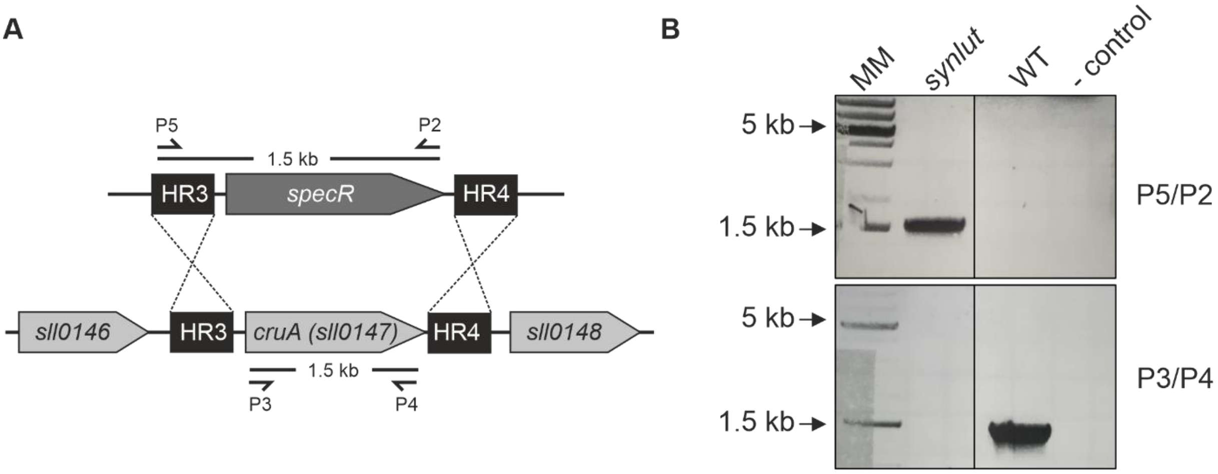

SUPPLEMENTARY FIGURE S1 | PCR analysis of the *synlut* strain and the wild type (WT) to prove full segregation using two different primer pairs (P5/P2, P3/P4, see **Supplementary Table S1** for further information. **(A)** Design of the *cruA* insertion cassette. A spectinomycin resistance-mediating gene was inserted between two sequences (HR3 and HR4) designed to mediate the deletion of the complete *cruA* coding sequence (*sll0147*). **(B)** Characterization of the *synlut* strain and its segregation status by PCR analyses. Primer-binding sites and product lengths are shown in panel **A**. MM: molecular marker. Full-length gels of panel **B** are presented in the **Supplementary Figure S2D**.

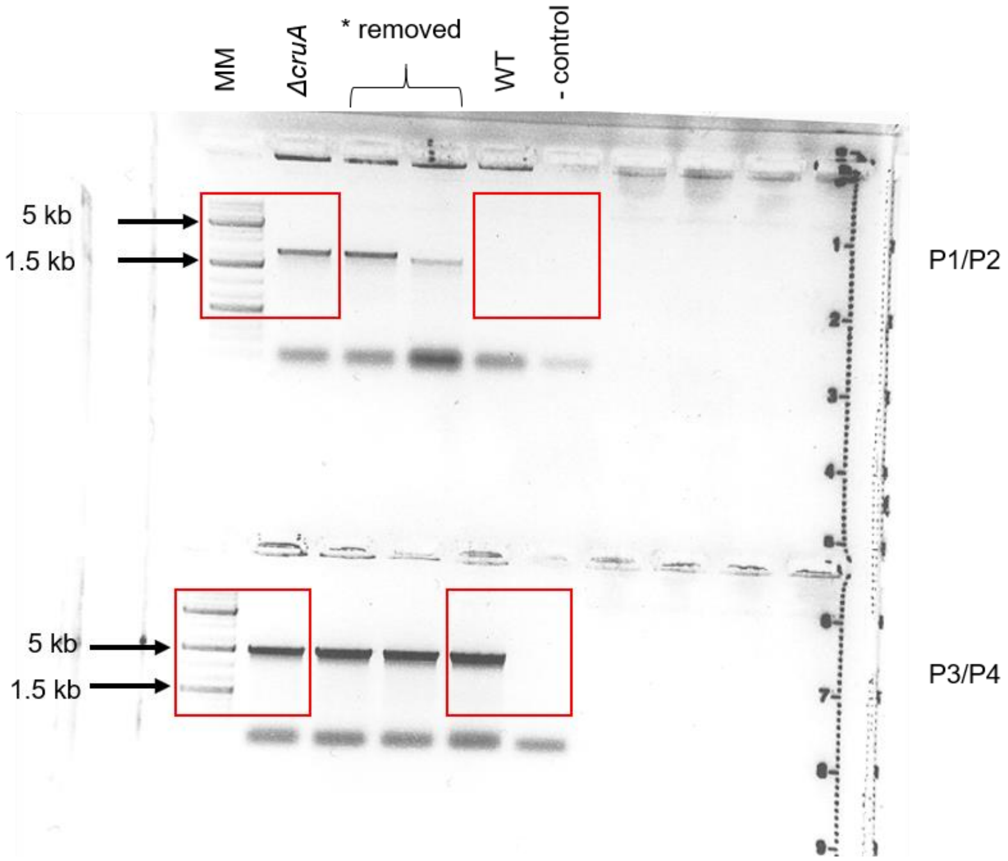

SUPPLEMENTARY FIGURE S2A | Raw data of manuscript **Figure 3B.** Isolation of *ΔcruA* knockdown strain. Characterization and the segregation status by PCR analyses. P1-P4: primers (see **Supplementary Table S3** for further information), MM: molecular marker. * not relevant for publication. Red boxes indicate parts used in the corresponding manuscript **Figure 3B**.

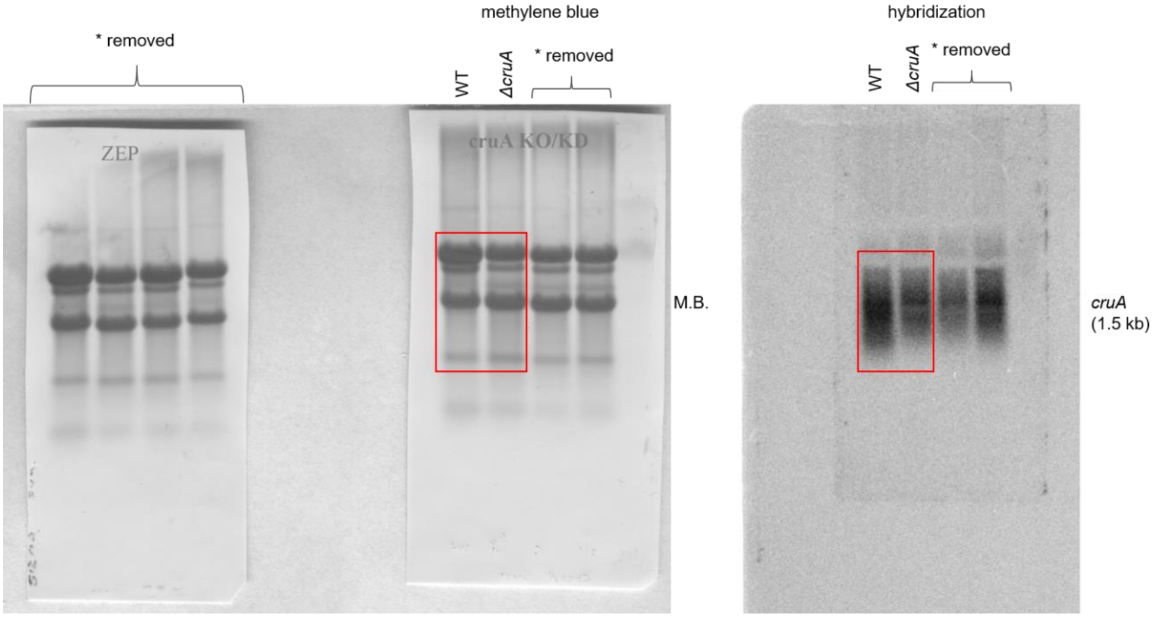

SUPPLEMENTARY FIGURE S2B | Raw data of manuscript **Figure 3C.** Isolation of *ΔcruA* knockdown strain. RNA gel-blot hybridization analyses with total RNA isolated from wild type (WT) and *ΔcruA*. After fractionation on a denaturing RNA gel and transfer to a nylon membrane, *cruA* transcripts were identified with radioactively labelled [α-^{32}P]dCTP DNA probes. Equal loading was checked by staining the nylon membranes with methylene blue solution (M.B.). * not relevant for publication. Red boxes indicate parts used in the corresponding manuscript **Figure 3C**.

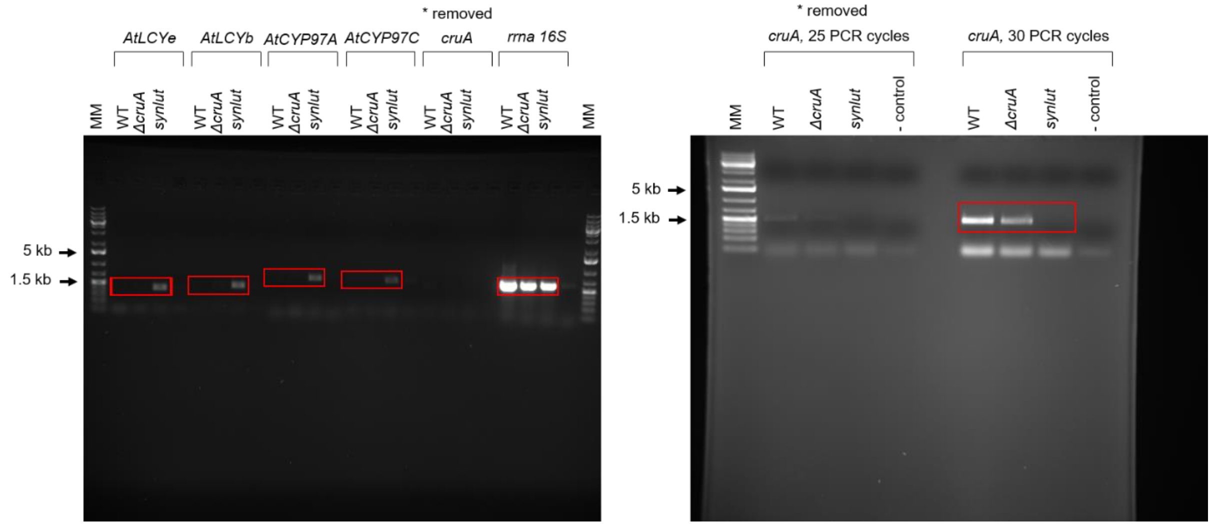

SUPPLEMENTARY FIGURE S2C | Raw data of manuscript **Figure 4D.** Whole-genome resequencing and expression analysis of *synlut*. Reverse transcription-PCR analyses of RNA samples isolated from WT, *ΔcruA* and *synlut* strains. *AtLCYe*, *AtLCYb*, *AtCYP97A*, *AtCYP97C* and *rrnA16S* cDNAs were amplified over 25 cycles, and the *cruA* cDNA was subjected to 30 PCR cycles, respectively. A PCR-based segregation analysis of *synlut* is provided in the **Supplementary Figure S1**. MM: molecular marker. * not relevant for publication. Red boxes indicate parts used in the manuscript **Figure 4D**.

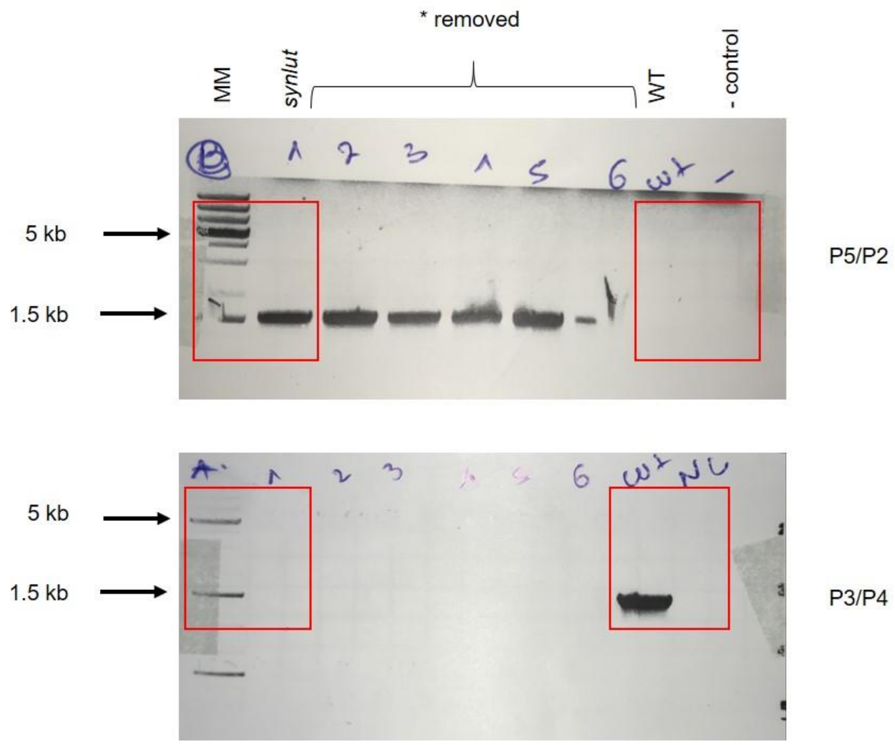

SUPPLEMENTARY FIGURE S2D | Raw data of **Supplementary Figure 1B**. PCR analysis of the *synlut* strain and the wild type (WT) to prove full segregation using two different primer pairs (P5/P2, P3/P4, see **Supplementary Table S1** for further information. MM: molecular marker. * not relevant for publication. Red boxes indicate parts used in the **Supplementary Figure S1B**.
